# Central Adrenal Insufficiency Unmasked After Pneumonia-Associated Asthma Exacerbation Leading to the Diagnosis of Rathke’s Cleft Cyst in an Elderly Woman: A Case Report

**DOI:** 10.7759/cureus.107114

**Published:** 2026-04-15

**Authors:** Natsumi Yamamoto, Ryuichi Ohta, Akira Yamasaki, Chiaki Sano

**Affiliations:** 1 Department of Community Care, Unnan City Hospital, Unnan, JPN; 2 Division of Respiratory Medicine and Rheumatology, Department of Multidisciplinary Internal Medicine, School of Medicine, Faculty of Medicine, Tottori University, Yonago, JPN; 3 Department of Community Medicine Management, Shimane University Faculty of Medicine, Izumo, JPN

**Keywords:** asthma exacerbation, central adrenal insufficiency, elderly patient, hyponatremia, panhypopituitarism, pituitary lesion, pneumonia, rathke’s cleft cyst, stress-induced adrenal insufficiency

## Abstract

Rathke’s cleft cyst (RCC) is a benign pituitary lesion that is often asymptomatic but can cause hypopituitarism, including central adrenal insufficiency, which may become clinically apparent under physiological stress. However, RCC presenting with adrenal insufficiency unmasked by acute respiratory illness is rare, particularly in elderly patients. We report a case of RCC diagnosed after adrenal insufficiency became evident following pneumonia-associated asthma exacerbation. An 89-year-old woman presented with fever, productive cough, and generalized fatigue and was diagnosed with pneumonia-triggered asthma exacerbation. Initial laboratory evaluation revealed hyponatremia (serum sodium 126 mEq/L). She was treated with bronchodilators, systemic corticosteroids, and antibiotics, resulting in prompt improvement of respiratory symptoms and normalization of serum sodium levels. After discontinuation of corticosteroids and discharge, she developed persistent fatigue, anorexia, and right eyelid ptosis. Brain magnetic resonance imaging revealed a 22-mm cystic lesion in the pituitary gland consistent with RCC. Endocrine evaluation demonstrated markedly reduced adrenocorticotropic hormone and cortisol levels, along with central hypothyroidism, hypogonadism, and growth hormone deficiency, confirming panhypopituitarism with central adrenal insufficiency. Hydrocortisone replacement therapy was initiated, leading to clinical stabilization. Surgical intervention was deferred due to advanced age and absence of visual field defects, and the patient was managed conservatively with hormone replacement and follow-up imaging. This case highlights that acute respiratory illness may precipitate overt adrenal insufficiency in patients with previously unrecognized RCC. Transient improvement with corticosteroids during acute treatment may mask the underlying endocrine disorder, delaying diagnosis. Persistent fatigue, hyponatremia, or neurological findings after resolution of respiratory illness should prompt evaluation for central adrenal insufficiency and pituitary pathology, as early recognition and hormone replacement are essential to prevent life-threatening complications.

## Introduction

Adrenal insufficiency is a clinical condition characterized by inadequate cortisol production, resulting in impaired physiological adaptation to stress and disturbances in water and electrolyte balance [1]. It is broadly classified into primary and central forms. In central adrenal insufficiency, dysfunction of the hypothalamus or pituitary gland results in insufficient secretion of adrenocorticotropic hormone (ACTH), thereby reducing cortisol production by the adrenal glands [2,3]. The adrenal glands are paired endocrine organs located above the kidneys and play an essential role in maintaining blood pressure, metabolism, and the stress response. The anterior pituitary regulates adrenal function through ACTH and also secretes other hormones, including growth hormone, gonadotropins, and thyroid-stimulating hormone (TSH) [2,3]. Therefore, pituitary disorders may result in multiple hormonal deficiencies and diverse clinical manifestations.

Rathke’s cleft cyst (RCC) is a benign cystic lesion arising from remnants of Rathke’s pouch during embryonic development and represents one of the more common mass lesions in the pituitary gland. Autopsy studies have reported a prevalence of approximately 10-20% [1]. Most cases remain asymptomatic and are incidentally detected by neuroimaging. However, some cysts enlarge and extend suprasellarly, causing neurological symptoms such as visual field defects and headache or leading to anterior pituitary dysfunction [2]. Hormonal deficiencies associated with RCC may involve growth hormone, gonadotropins, TSH, and ACTH. Among these, ACTH deficiency leading to central adrenal insufficiency is particularly important because delayed diagnosis can be life-threatening [2,3].

The clinical manifestations of adrenal insufficiency are often nonspecific and include fatigue, anorexia, weight loss, hypotension, and hyponatremia, which can easily be misattributed to aging or comorbidities in elderly patients, thereby delaying diagnosis [2,3]. Adrenal insufficiency often becomes clinically evident under stressful conditions such as infection, surgery, or trauma. In particular, patients with latent adrenal insufficiency may experience rapid clinical deterioration when exposed to acute stress without adequate hormonal compensation [4,5].

In primary adrenal insufficiency, hyponatremia is reported in approximately 85-90% of patients [4]. In central adrenal insufficiency, aldosterone secretion is generally preserved because it is primarily regulated by the renin-angiotensin-aldosterone system; therefore, hyperkalemia is uncommon. However, impaired free water clearance and increased vasopressin activity may cause dilutional hyponatremia [5]. Previous studies have shown that 2.7-3.8% of patients with euvolemic hyponatremia had previously unrecognized adrenal insufficiency [6] and that 1.7% of patients with severe hyponatremia (Na ≤120 mmol/L) were ultimately diagnosed with adrenal insufficiency as the underlying cause [7]. These findings highlight that even in central adrenal insufficiency, hyponatremia can serve as an important diagnostic clue.

The present case is noteworthy because relative adrenal insufficiency became apparent following pneumonia-associated asthma exacerbation, and further evaluation led to the diagnosis of RCC. Reports of RCC presenting with hypopituitarism exist [2,3]. However, cases in which central adrenal insufficiency was unmasked by a respiratory event and resulted in the diagnosis of RCC are exceedingly rare. This case underscores the importance of considering central adrenal insufficiency and pituitary lesions, including RCC, in the differential diagnosis of persistent fatigue and hyponatremia in elderly patients after acute respiratory illness.

## Case presentation

An 89-year-old woman presented with a three-day history of fever, productive cough, and generalized fatigue. Her past medical history included bronchial asthma, hypertension, dyslipidemia, gastroesophageal reflux disease, and liver cirrhosis due to hepatitis C virus infection. No previously recognized endocrine disorder, chronic hypotension, or unexplained recurrent hyponatremia had been documented before this admission. Her medications included theophylline 200 mg daily, montelukast 10 mg daily, benidipine 2 mg daily, valsartan 40 mg daily, rosuvastatin 5 mg daily, and esomeprazole 10 mg daily.

On arrival, her vital signs were as follows: blood pressure 185/56 mmHg, heart rate 106 beats/min, body temperature 37.6°C, respiratory rate 24 breaths/min, and SpO₂ 97% on room air. On physical examination, wheezing was audible in both inspiratory and expiratory phases. Neurologically, right eyelid ptosis was noted, but ocular movements were normal, and there was no facial weakness. No leg edema was observed.

Initial laboratory tests showed hyponatremia and anemia (Table 1).

**Table 1:** Initial laboratory data of the patient

Parameters	Level	Reference
White blood cells	5.00	3.5-9.1 × 10^3^/μL
Neutrophils	71.1	44.0-72.0%
Lymphocytes	13.9	18.0-59.0%
Hemoglobin	9.8	11.3-15.2 g/dL
Hematocrit	30.4	33.4-44.9%
Mean corpuscular volume	68.2	79.0-100.0 fl
Platelets	15.2	13.0-36.9 × 10^4^/μL
Total protein	7.9	6.5-8.3 g/dL
Albumin	4.4	3.8-5.3 g/dL
Total bilirubin	0.9	0.2-1.2 mg/dL
Aspartate aminotransferase	28	8-38 IU/L
Alanine aminotransferase	62	4-43 IU/L
Lactate dehydrogenase	231	121-245 U/L
Blood urea nitrogen	13.0	8-20 mg/dL
Creatinine	1.13	0.40-1.10 mg/dL
Serum Na	126	135-150 mEq/L
Serum K	3.8	3.5-5.3 mEq/L
Serum Cl	91	98-110 mEq/L
Ferritin	40.4	14.4-303.7 ng/mL
Urine test	-	-
Leukocyte	Negative	Negative
Protein	Negative	Negative
Blood	Negative	Negative
Ketone	Negative	Negative

**Figure 1:**
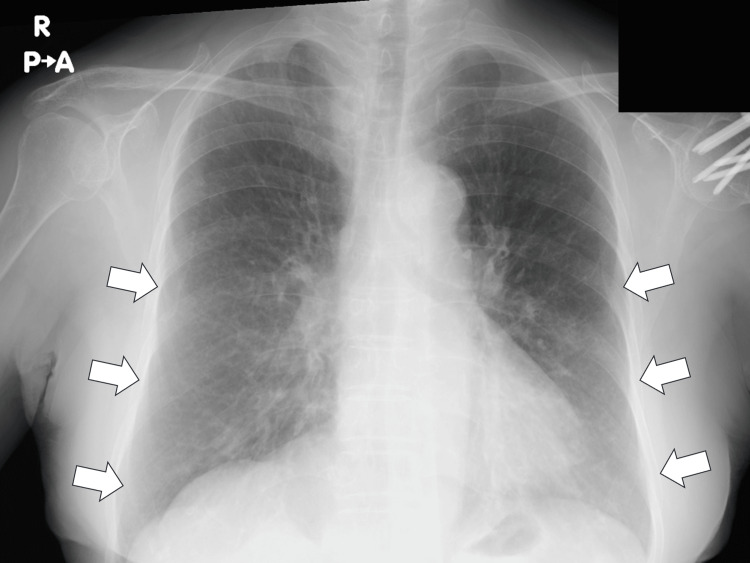
Chest X-ray demonstrating patchy ground-glass opacities in the bilateral middle lung fields (white arrows)

Chest X-ray demonstrated patchy ground-glass opacities in the bilateral middle lung fields (Figure 1).

Echocardiography revealed a left ventricular ejection fraction >50%, mild tricuspid regurgitation, and an inferior vena cava diameter of 22/7 mm. Electrocardiography showed sinus rhythm without ST-T abnormalities.

The patient was diagnosed with a moderate asthma attack triggered by pneumonia. Treatment was initiated with inhaled salbutamol, oral prednisolone 30 mg every eight hours, inhaled vilanterol/fluticasone, and intravenous ceftriaxone 2 g/day for five days. Her respiratory symptoms resolved promptly, and serum sodium normalized (136 mEq/L) during systemic corticosteroid treatment. Prednisolone was then discontinued, and she was discharged home a few days later.

**Figure 2:**
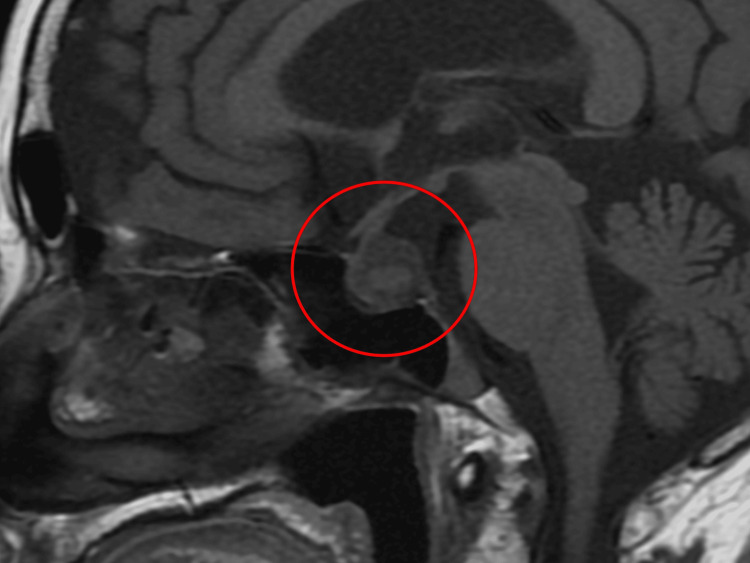
Brain contrast-enhanced magnetic resonance imaging (MRI) revealing a 22-mm cystic lesion in the pituitary gland (red circle)

At follow-up seven days after discharge, although her respiratory symptoms had improved, she continued to experience anorexia and generalized fatigue. In addition, the right eyelid ptosis noted on admission persisted. Because these symptoms were disproportionate to the improvement in pneumonia and asthma, further evaluation was performed, including brain contrast-enhanced magnetic resonance imaging (MRI), which revealed a 22-mm cystic lesion in the pituitary gland (Figure 2).

**Figure 3:**
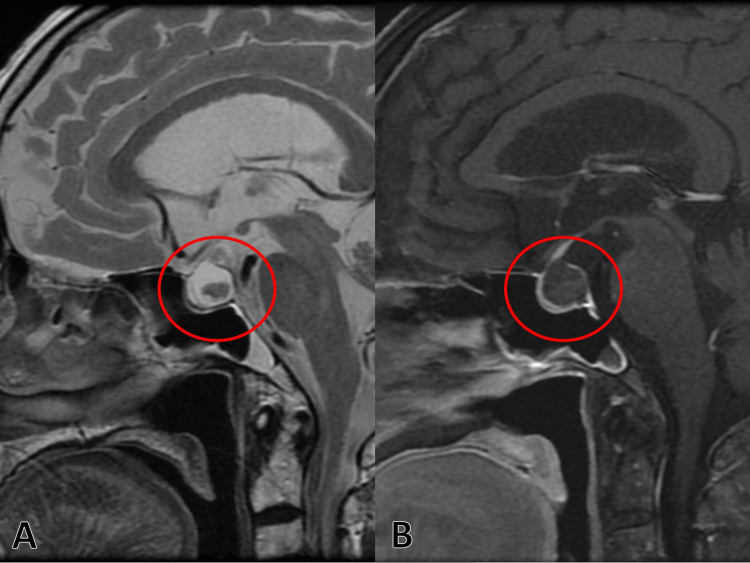
Brain contrast-enhanced magnetic resonance imaging (MRI) with T2-weighted imaging showing high signal intensity with internal low-signal areas (A) and no contrast enhancement (B), consistent with a Rathke’s cleft cyst (red circles)

On T2-weighted imaging, the lesion showed high signal intensity with internal low-signal areas and no contrast enhancement, consistent with an RCC (Figure 3).

After discontinuation of systemic corticosteroids, endocrine evaluation was performed because of persistent fatigue, anorexia, hyponatremia, and the pituitary lesion on MRI. Hormonal studies demonstrated central adrenal insufficiency, hypothyroidism, and hypogonadism, leading to the diagnosis of panhypopituitarism associated with a pituitary cystic lesion (Table 2).

**Table 2:** Additional laboratory data of the patient Abbreviations: ACTH, adrenocorticotropic hormone; ADH, antidiuretic hormone; FSH, follicle-stimulating hormone; Free T3, free triiodothyronine; Free T4, free thyroxine; GH, growth hormone; LH, luteinizing hormone; TSH, thyroid-stimulating hormone

Parameters	Level	Reference
ACTH	<1.5	7.2-63.3 pg/mL
Cortisol	<1.0	4.5-21.1 μg/dL
TSH	0.05	μU/mL
Free T3	2.1	1.88-3.18 pg/mL
Free T4	0.8	0.7-1.48 ng/dL
LH	<1.0	mIU/mL
FSH	0.90	mIU/mL
GH	0.11	0.13-9.88 ng/mL
ADH	0.9	1-5 pg/mL

Hydrocortisone replacement therapy (10 mg/day) was initiated. Given her advanced age and absence of visual field defects, surgical intervention was not performed. She was managed conservatively with continued hormone replacement in the outpatient department.

## Discussion

In this case, an 89-year-old woman developed overt central adrenal insufficiency following pneumonia-associated asthma exacerbation, which ultimately led to the diagnosis of RCC causing panhypopituitarism. Although her respiratory symptoms and hyponatremia initially improved with systemic corticosteroid therapy, persistent fatigue and neurological findings after steroid withdrawal prompted further evaluation. Brain MRI revealed a 22-mm cystic pituitary lesion consistent with RCC, and endocrine testing confirmed severe ACTH deficiency with multiple anterior pituitary hormone deficiencies. This case illustrates how acute respiratory stress and transient corticosteroid administration can unmask and simultaneously obscure underlying central adrenal insufficiency.

RCC is detected in approximately 10-20% of autopsy studies, yet most lesions remain asymptomatic throughout life [8]. Symptomatic RCC may cause mass effect or varying degrees of hypopituitarism, depending on cyst size and compression of normal pituitary tissue [2,8]. Among the possible hormonal deficits, ACTH deficiency is particularly critical due to its potential to precipitate life-threatening adrenal crisis [4,8,9]. While hypopituitarism secondary to RCC has been reported [2,8], cases in which central adrenal insufficiency becomes clinically apparent during an acute respiratory illness are exceedingly rare, especially in very elderly patients.

Adrenal insufficiency frequently manifests under physiological stress, such as infection, trauma, or surgery [10]. In patients with latent ACTH deficiency, acute illness may exceed residual adrenal reserve, resulting in overt clinical deterioration [10]. In the present case, pneumonia and asthma exacerbation likely acted as stressors, revealing insufficient endogenous cortisol production. Importantly, the administration of systemic corticosteroids for asthma temporarily compensated for cortisol deficiency, normalizing serum sodium and masking the underlying endocrine disorder [11,12]. After discontinuation of steroids, symptoms recurred, highlighting the phenomenon of “masked adrenal insufficiency” in patients treated empirically with glucocorticoids for non-endocrine indications [13].

Hyponatremia served as an important early diagnostic clue. Although hyperkalemia is uncommon in central adrenal insufficiency due to preserved mineralocorticoid function, impaired free water clearance and increased vasopressin activity can lead to dilutional hyponatremia [6]. Previous studies have shown that adrenal insufficiency accounts for a small but clinically significant proportion of euvolemic or severe hyponatremia cases [14]. In elderly patients, nonspecific symptoms such as fatigue, anorexia, and mild cognitive or neurological changes are often attributed to aging or comorbidities [15]. In this context, persistent or unexplained hyponatremia after resolution of infection should prompt evaluation of adrenal function, even when initial improvement is observed during corticosteroid therapy.

Another notable feature in this case was unilateral ptosis at initial presentation. Although visual field defects are the classic neurological manifestation of sellar mass lesions, subtle cranial nerve-related findings may precede overt visual impairment [16]. The coexistence of neurological signs and electrolyte abnormalities should therefore raise suspicion for central pathology.

Management decisions in elderly patients with RCC require individualized risk-benefit assessment. Surgical intervention is generally indicated for symptomatic mass effect or progressive visual compromise [8]. However, in advanced age and in the absence of visual field defects, conservative management with hormone replacement and radiological follow-up is a reasonable strategy. In this patient, hydrocortisone replacement resulted in clinical stabilization without immediate need for surgical intervention.

This case carries several important clinical implications in general medicine. First, acute respiratory illness may precipitate overt adrenal insufficiency in patients with previously unrecognized pituitary dysfunction. Second, transient improvement during systemic steroid therapy can delay diagnosis by masking endocrine insufficiency. Third, persistent fatigue, anorexia, hyponatremia, or subtle neurological findings in elderly patients should prompt consideration of central adrenal insufficiency and pituitary imaging. Early recognition and appropriate hormone replacement are essential to prevent adrenal crisis and improve outcomes in older patients with vague symptoms dealt by general physicians [17].

## Conclusions

In this case, an elderly woman developed overt central adrenal insufficiency following pneumonia-associated asthma exacerbation, which ultimately led to the diagnosis of an RCC. Although her acute respiratory symptoms and hyponatremia improved with systemic corticosteroid therapy, the underlying endocrine disorder was temporarily masked. Recurrence of fatigue after steroid withdrawal prompted further evaluation, revealing panhypopituitarism due to RCC. Adrenal insufficiency frequently becomes clinically apparent under physiological stress such as infection, particularly in patients with previously unrecognized pituitary dysfunction. In elderly individuals, nonspecific symptoms - including fatigue, anorexia, and hyponatremia - may delay diagnosis. While RCC-related hypopituitarism has been described, presentation triggered by an asthma exacerbation is exceedingly uncommon. Clinicians should consider central adrenal insufficiency and pituitary pathology in elderly patients with persistent fatigue or unexplained hyponatremia following acute respiratory illness. Early recognition and appropriate hormone replacement are essential to prevent potentially life-threatening complications.
